# Butterfly phenology in Mediterranean mountains using space‐for‐time substitution

**DOI:** 10.1002/ece3.5951

**Published:** 2020-01-02

**Authors:** Konstantina Zografou, Andrea Grill, Robert J. Wilson, John M. Halley, George C. Adamidis, Vassiliki Kati

**Affiliations:** ^1^ Institute of Ecology and Evolution University of Bern Bern Switzerland; ^2^ Department of Biological Applications and Technology University of Ioannina Ioannina Greece; ^3^ Museo Nacional de Ciencias Naturales (MNCN‐CSIC) Madrid Spain

**Keywords:** changing climate, developmental delay, elevational gradient, emergence time, flight period

## Abstract

Inferring species' responses to climate change in the absence of long‐term time series data is a challenge, but can be achieved by substituting space for time. For example, thermal elevational gradients represent suitable proxies to study phenological responses to warming. We used butterfly data from two Mediterranean mountain areas to test whether mean dates of appearance of communities and individual species show a delay with increasing altitude, and an accompanying shortening in the duration of flight periods. We found a 14‐day delay in the mean date of appearance per kilometer increase in altitude for butterfly communities overall, and an average 23‐day shift for 26 selected species, alongside average summer temperature lapse rates of 3°C per km. At higher elevations, there was a shortening of the flight period for the community of 3 days/km, with an 8.8‐day average decline per km for individual species. Rates of phenological delay differed significantly between the two mountain ranges, although this did not seem to result from the respective temperature lapse rates. These results suggest that climate warming could lead to advanced and lengthened flight periods for Mediterranean mountain butterfly communities. However, although multivoltine species showed the expected response of delayed and shortened flight periods at higher elevations, univoltine species showed more pronounced delays in terms of species appearance. Hence, while projections of overall community responses to climate change may benefit from space‐for‐time substitutions, understanding species‐specific responses to local features of habitat and climate may be needed to accurately predict the effects of climate change on phenology.

## Graphical abstract

Substituting space for time showed novel insights on how butterfly community and individual species respond to a changing environment along an altitudinal gradient in two mountainous areas, in the Mediterranean basin. A general delay in the time of appearance and a progressive shortening in the duration of the flight period with increased altitude was supported, but not the unimodal response of species with different number of generations and trophic preferences. This study is a roadmap for analyzing short‐term data in the light of the ongoing climate change, when elevational gradient is available.

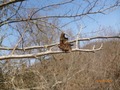

## 1 INTRODUCTION

Phenology, the timing of seasonal events in the life cycles of fauna and flora, has been identified as an important metric to track ecological responses to climate change (Altermatt, 2012; Cohen, Lajeunesse, & Rohr, 2018; Diamond, Frame, Martin, & Buckley, 2011; Forister & Shapiro, 2003; Parmesan & Yohe, 2003; Stefanescu, Penuelas, & Filella, 2003; Wilson et al., 2005; Zografou et al., 2015). There is evidence for both communities and individual species of butterfly that adults emerge earlier as the climate warms (Dell, Sparks, & Dennis, 2005; Lopez‐Villalta, 2010; Stefanescu et al., 2003; Wilson et al., 2005) and that flight periods have become longer (Menzel et al., 2006). These phenological changes as the climate warms could result from adaptations (Schilthuizen & Kellermann, 2014) including increased numbers of generations in multivoltine species (Altermatt, 2010), or the facilitation of another generation in principally univoltine species (Fischer & Fiedler, 2002).

The most robust forecasts of phenological responses to climate change combine high‐quality monitoring data collected over an extended time period (de Arce Crespo & Gutiérrez, 2011; Banet & Trexler, 2013) with an identified dependence of a phenological trait on reliable environmental cues (Reed, Waples, Schindler, Hard, & Kinnison, 2010). Detailed phenological time series have been analyzed for butterflies in northern and central Europe (Altermatt, 2012; Roy & Sparks, 2000; Van Strien, Plantenga, Soldaat, Swaay, & WallisDeVries, 2008) and elsewhere (Brooks et al., 2017; Diamond et al., 2011), but many other regions lack long enough time series of monitoring data. Populations of species respond differently to climatic conditions in different parts of their geographic ranges (Mills Simon et al., 2017; Scranton & Amarasekare, 2017), so evidence of how phenology varies with climate in a variety of different locations (including relatively unexplored areas) can increase understanding both of individual species responses and of the likely effects of climate change on the phenology of a more representative range of ecological communities.

When no long‐term data are available, an alternative approach to studying phenology is to substitute space for time, by assuming that the spatial relationship between an environmental factor (e.g., elevation) and a phenological response (e.g., time of appearance) can be used as a proxy for the temporal relationship (Banet & Trexler, 2013). In this way, studies investigating how phenotypic traits change along latitudinal or elevational gradients can contribute to the prediction of species responses to climate change (de Arce Crespo & Gutiérrez, 2011; Gutiérrez & Menéndez, 1998; Hodkinson, 2005; Leingärtner, Krauss, & Steffan‐Dewenter, 2014; Merrill et al., 2008). However, space‐for‐time substitution can become less valid at certain spatiotemporal scales (Blois, Williams, Fitzpatrick, Jackson, & Ferrier, 2013) or lead to underestimations of changes in diversity (França et al., 2016) especially under the pressure of a changing environment (Damgaard, 2019). Therefore, the implicit use of space‐for‐time substitution should be treated with caution in modeling community responses to climate change.

Information on species' ecological and life‐history traits has also been used to further understanding of interspecific variation in phenological responses to climate change (Diamond et al., 2011; Kharouba, Paquette, Kerr, & Vellend, 2014; Leingärtner et al., 2014). For example, species whose flight periods occur earlier in the year and less mobile species appear more sensitive to temperature variation than late flying or more mobile species (Kharouba et al., 2014), and multivoltine species may be more able to respond to warming by increasing the frequency of their annual generations (Altermatt, 2009, 2010). Although earlier emergence dates and increased numbers of generations have been widely documented, it has also been shown that some insects could be negatively affected by warmer climates. If juvenile stages complete development in late summer instead of entering the overwintering stage, a lack of sufficient time and suitable conditions to breed could lead to population declines (“the lost generation hypothesis”) (Glazaczow, Orwin, & Bogdziewicz, 2016; Van Dyck, Bonte, Puls, Gotthard, & Maes, 2015; van der Kolk, WallisDeVries, & Vliet, 2016). Also for butterflies performing a photoperiodically induced summer dormancy, like Mediterranean *Maniola* butterflies (Van Dyck et al., 2015), climate warming might have negative effects on populations. If the summer drought became extended and the butterfly deposited her eggs before the onset of vegetation regrowth, triggered by a shortened photoperiod at the beginning of autumn, the young larvae would have no suitable fresh grasses to feed on and starve to death. Other negative consequences of climate change could include phenological mismatches between trophically interacting species such as butterflies and their host plants (Bale et al., 2002; Parmesan & Yohe, 2003; Visser & Both, 2005). Overall, climate change is responsible for well‐studied phenological shifts, but their magnitude and direction can largely vary even between species inhabiting the same latitude (Diez et al., 2012). For these reasons, it is important to assess the effect of climate change on phenology from a comprehensive range of environments and ecological communities.

Elevational gradients are potentially useful space‐for‐time proxies because they combine significant variation in temperature over short geographic distances (Körner, 2007) with minimal variability in photoperiod (Fielding, Whittaker, Butterfield, & Coulson, 1999; Hodkinson, 2005). In addition, microclimate and habitat conditions (including vegetation structure and canopy cover) vary over elevational gradients (Suggitt et al., 2011) and can buffer ecological communities against coarse‐scale trends and patterns in climate change (Gillingham, Huntley, Kunin, & Thomas, 2012; Suggitt et al., 2012). Therefore, phenology can vary markedly over elevational gradients but also within an altitudinal belt depending on habitat type, and Altermatt (2012) showed that the seasonal appearance of butterflies is influenced by both of these variables. Testing local effects of elevation and habitat on phenology using space‐for‐time assumption could be a valid approach to understanding and predicting ecological responses to climate change (Banet & Trexler, 2013; Hodgson et al., 2011; Leingärtner et al., 2014). Although this method has some caveats (e.g., it cannot track year‐to‐year changes in species phenology), it can, however, serve as a short‐term “tracking device” that mimics the longer seasons and milder winters that are expected as the climate warms (EEA, 2017; van der Wiel, Kapnick, & Vecchi, 2017).

In general, increasing elevation is expected to influence species' phenology by shortening the annual activity window, forcing stages in the life cycle to appear later while maintaining synchrony with resources and suitable environmental conditions (Brown & Lomolino, 1998; Despland, Humire, & Martín, 2012; Hodkinson, 2005). There is much evidence that temperate butterflies become active later annually at higher elevations (de Arce Crespo & Gutiérrez, 2011; Illán, Gutiérrez, Díez, & Wilson, 2012; Merrill et al., 2008; Shapiro, 1975). In addition, there is evidence that climate warming has led to both earlier appearance and extension of the flight period at high elevations (Konvička, Beneš, Čížek, Kuras, & Klečková, 2016). In this paper, using space‐for‐time inferences we frame phenological responses to climate change in a biota which lacks long‐term data. By examining the phenology of butterfly communities in two mountainous areas in Greece, across different elevations and habitat types, we investigate the following research questions:

1. Is there a delay in the appearance of species and a progressive shortening of the flight period at higher elevations? We predict that phenological windows of activity will be shorter, better synchronized and delayed with elevation (Despland et al., 2012; Illán et al., 2012).
2. Are altitudinal patterns of butterfly phenology consistent with the temperature lapse rates recorded for each mountain system? We expect to find steeper changes in emergence patterns when temperature lapse rate is steep and therefore climatic differences between elevations are more pronounced.
3. Do phenological patterns differ among different habitat types (agricultural areas, grassland, and forest)? We expect that phenology may vary with elevation at a different rate in different habitat types (Zografou et al., 2015). For example, microclimates in forests that have a denser canopy compared to open habitats are less influenced by direct radiation (Scherrer & Körner, 2011), potentially leading to longer delays in emergence compared to open habitats (e.g., grasslands).
4. Do the responses of individual species follow consistent patterns with elevation in terms of time of the appearance and duration of the flight period? We expect that univoltine species will show less pronounced altitudinal variation in phenology as a result of lesser adaptability compared with multivoltine species.

## 2 MATERIALS AND METHODS

### 2.1 Study system

Our study area consisted of two mountain regions that differ in geographic position, areal extent, biome, climate type, and topography. The Rodopi mountain chain (Rodopi hereafter: long. 24° 23′, lat. 41°23′; maximum elevation 2,323 m) is located in NE Greece, whereas Grammos (long. 20°50′, lat. 40°21′; maximum elevation 2,520 m) is located in NW Greece (Figure S1, Table S1, but see also Zografou, Wilson, Halley, Tzirkalli and Kati (2017) for detailed descriptions). Both systems share a low human population density and associated low‐intensity human activities, as well as high coverage by protected areas of the Natura 2000 network. The climate in Rodopi is at the transition between Mediterranean and a continental climate (Mavromatis, 1980) with a mean annual temperature of 11.4°C and mean annual precipitation of 1,200 mm, while the climate in Grammos is humid continental (Korakis, 2002) with a mean annual temperature of 8–12°C and mean annual rainfall of 1,500 mm.

### 2.2 Butterfly sampling

Butterflies were recorded at 41 sites in Rodopi and 26 in Grammos. The minimum distance between nearest neighboring sites was approximately 2 km (*SD* ± 0.5) so that each site effectively represents an independent sampling unit. The lack of spatial autocorrelation between nearby sites was verified in terms of alpha and beta components of diversity in a previous study where we investigated diversity patterns of butterflies and Orthoptera across different spatial scales (Zografou et al., 2017). Each mountain was partitioned into four elevation zones (0–500 m, 501–1,000 m, 1,001–1,500 m, and 1,501–2,000 m) and each zone contained sites representing the three dominant habitats found in the study system (agricultural fields, grasslands, and forests), with the exception that agricultural areas were not present above 1,500 m (Figure S1, Table S1). Permanent transect routes were established at two to six sites representative of each habitat type per altitudinal zone in each mountain range, recording geographic location (UTM) and elevation (m) using a hand‐held GPS unit. On each site visit, the transect was walked at a steady pace under weather conditions that were suitable for butterfly activity (Pollard & Yates, 1993) recording all butterflies observed along a standardized length and width of 300 × 5 m. Butterflies were captured with the help of hand net, identified in situ, and when necessary photographic material was also collected for confirming identification in the laboratory. We visited each site five times from April until August 2012 (Rodopi) and four times from May until August 2013 (Grammos—no sampling conducted in April due to unsuitable weather). Each transect was walked with a maximum sampling interval of 20 days between visits: This was the minimum interval which was feasible for a single field observer to achieve, given unpredictable weather and occasionally inaccessible sites particularly at higher elevations.

### 2.3 Phenological descriptors

The timing and duration of flight periods were calculated to describe species' phenology along the altitudinal gradient. For each species, the timing of flight period was summarized per site as the weighted mean date (hereafter mean date) by summing counts per visit, according to the formula:

$$\text{Mean date}\;=\;\sum_{\text{Visits}}\frac{\left(\text{Individuals per visit})\;\times \;(\text{Date of visit}\right)}{\text{Total number of individuals}}$$

Date was estimated in Julian dates, and data were summed for each species across all visits (1 January = 1, 1 February = 32, etc.). Mean date is a commonly used descriptor in phenological studies for butterflies and considered to be more reliable than other phenological measures such as the first day of adult appearance (Van Strien et al., 2008). In addition, as the occurrence of butterfly individuals in temperate species follows an approximately normal frequency distribution (Arce Crespo & Gutierrez, 2011), the use of mean date considers to be a safe approach (Moussus, Julliard, & Jiguet, 2010). We acknowledge the lack of multiple visits per month (e.g., weekly) but we emphasize that the main purpose is to examine relative differences in the degree of phenological shift, rather than to get unbiased estimates of the extent to which phenology change. We also calculated the duration of flight period as the standard deviation about the mean date (Brakefield, 1987). At the community level, we used all species for which the estimation of the two phenological descriptors could be generated (87) and when comparing the mean flight dates of butterfly communities between the two mountains and across different habitat types, only species present in both mountains (87) or all habitat types (33) were considered.

At species level, we analyzed data for 26 species that (a) were recorded in more than three sites with at least two records per site, (b) do not overwinter as adults (e.g., *Inachis io*, *Gonepteryx rhamni*) or fly in early spring (e.g., *Anthocharis cardamines*, *Callophrys rubi*) as numbers of these species could be underestimated due to the dates when we began sampling, and (c) were not long‐distance migrants (*Colias crocea*, *Issoria lathonia*, *Pieris brassicae*) as appearance in the mountains will be biased by population situations elsewhere. Information on overwintering stage and voltinism was extracted from published sources (Pamperis & Stavridis, 2009; Tolman & Lewington, 1997). To investigate interspecific relationships of species phenology with sample size and with elevational range, we also calculated the following measures for each species: the number of sites where the species was present, the minimum elevation, the maximum elevation, and the elevational range (maximum–minimum).

### 2.4 Data analysis

For our first hypothesis, we investigated variation with elevation in the timing and duration of the flight period. We carried out linear‐mixed models where the mean date and standard deviation about the mean date were modeled as a function of altitude, mountain, and habitat. In addition, species were included as a random effect. Models were validated by checking for homoscedasticity and normality of the residuals (Zuur, Ieno, Walker, Saveliev, & Smith, 2009), and in all cases, diagnostic graphs showed that model assumptions were met (Figure S2). For these models, altitude slope represented the delay (in days/km).

To investigate our second research question, we evaluated whether butterfly assemblages occurring in Rodopi have greater elevational delays in emergence compared with their counterparts in Grammos (considering species common to both mountain ranges), as a result of the different rates of climatic variation with elevation between the two mountains. We did this using standardized major axis (SMA) analysis. SMA is especially suitable when the prime interest is to inspect the slopes to see how each pair of variables is related to each other, rather than predicting Y (phenological descriptor) from X (elevation). In addition, SMA is a slope‐fitting technique that shows how one variable scales against another, and slopes are fitted via a permutation test by minimizing the residual variance in X and Y dimensions simultaneously rather than Y alone (Domínguez et al., 2012; Falster & Westoby, 2005) resulting thus in a less biased outcome compared to traditional approaches such as ANCOVA (Warton, Wright, Falster, & Westoby, 2006). As a result, the sampling error which in our case is derived by the high topographic variability of mountain ranges can be minimized and biased slopes avoided (Legendre, 2001). Although SMA has been recommended particularly for allometric studies (Warton, Wright, & Wang, 2012), it can also be applied to ecological responses to environmental variables in the context of climate change (Zografou, 2015). Mountain was used as the grouping factor, and we discarded the first sampling in Rodopi (April 2012) in order to ensure that data (of four visits between May and August) were comparable between the two mountain ranges.

For the third research question, we used the same approach and tested whether butterfly assemblages that occur in forests have longer phenological delays (steeper slopes) with elevation compared to their counterparts in grasslands or agriculture areas.

To investigate our last research question regarding variation in the phenology of individual species with elevation, we ran general linear models for the 26 selected species, using the regression slope to estimate the delay in days/m. To account for between mountain and habitat variation, both terms were included in the models and *p* values were corrected using Benjamini and Hochberg (1995) adjustment method. We also tested whether the elevational delay was related to the number of sites where a species was present and the species' elevational range. Species that are present in more sites or species with wider elevational ranges may be expected to have longer delays compared to those whose distributions are limited to fewer sites or high elevations only, because the latter species may exhibit flight periods synchronized within a narrow phenological window, for example, avoiding the risk of unfavorable weather conditions in late summer (Illán et al., 2012).

The analyses were conducted in R (version 3.3.1; R Core Team, 2014), specifically using lm function (Chambers, 1992; Wilkinson & Rogers, 1973) for general linear models and lme4 package for mixed‐effects models (Bates, Mächler, Bolker, & Walker, 2015), and the SMATR 3 package (Warton, Duursma, Falster, & Taskinen, 2012) for SMA analysis.

To visualize our general linear‐ and mixed‐effects models with partial residual plots, we extracted adjusted data using the “visreg” function in the “VISREG” package (Breheny & Burchett, 2017). Finally, the “ggplot” function of the “ggplot2” package (Wickham, 2016) was used and ggplot2 library for the graphical representation of our results.

### 2.5 Temperature lapse rate

We collected temperature data at each site using a Hobo data logger, to determine the gradient in seasonal temperature over elevation (lapse rate) overall and for each mountain. The logger was placed in full shade at the beginning of each transect walk and recorded temperature (°C) each minute until the end of the sampling event (approximately 90 min). We used the same logger to record temperature during each site visit. A mean value for temperature at each site was calculated using all temperature measurements from each visit between May and August. Lapse rate was calculated by regressing the mean temperature per site against elevation.

## 3 RESULTS

### 3.1 Phenological patterns at the community level

**Figure 1:**
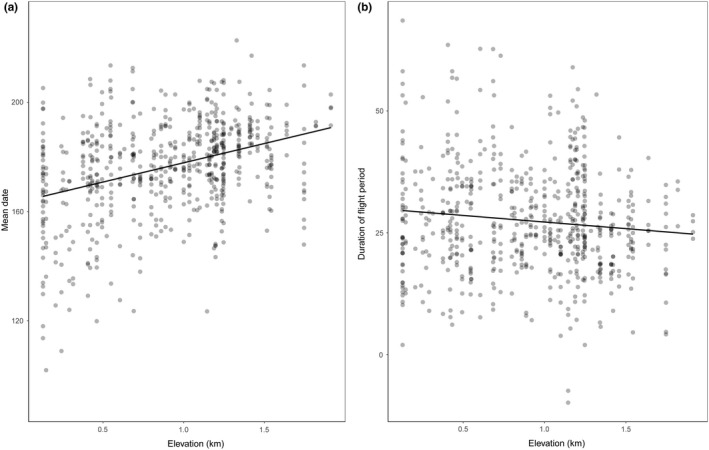
Partial residuals and prediction lines showing effects of elevation on (a) mean date (days since 1 January, 1 January = 1) and (b) duration of the flight period (standard deviation about the mean date). Dots correspond to the mean date of a species per sampling site

We found a positive and significant relationship between the variables “mean date” and “elevation” for the whole species pool (i.e., butterflies that occur in both mountains) (*p* < .001; mean date = 169 + 14.11 × elevation), a delay of 14 days for every kilometer increase in elevation. There was considerable variation in mean flight date around this main pattern, with a 112‐day interval between mean flight date at the earliest low‐elevation site and the latest high‐elevation site (Figure 1a). The relationship between the duration of flight period and elevation was also significant (*p* = .01; duration = 23.20–2.71 × elevation). The negative slope indicates a shortening of the duration of the butterfly flight period with increasing elevation of approximately 3 days per kilometer (Figure 1b).

### 3.2 Phenological patterns between mountains and across habitats

**Figure 2:**
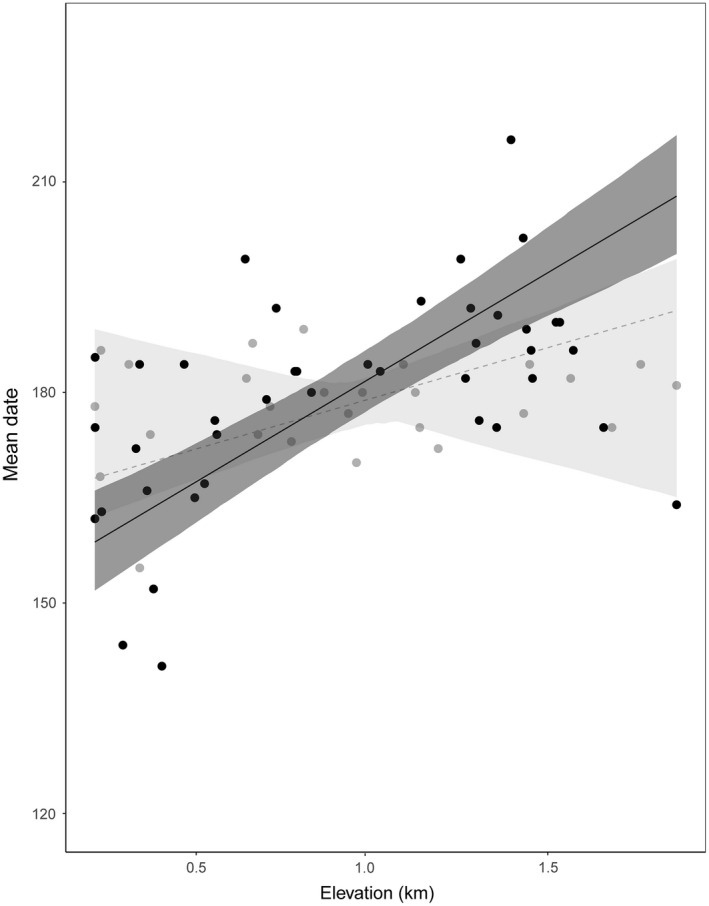
Variation in relationships between mean date and elevation for the two mountains. Gray line and dots correspond to Grammos and black to Rodopi. Only butterfly species present in both mountains were considered for the calculation of the mean date. Each dot corresponds to a sampling site (total number of sites, *n* = 67), and dotted lines refer to nonsignificant regression lines

The relationship between mean date and elevation differed between mountains (LR test: 6.95, *p* = .007, *n* = 67) indicating a different rate at which butterfly assemblages delayed their appearance date with elevation (Figure 2). Both ecoregions had positive slopes, but Rodopi showed a steeper slope, indicating a bigger delay of butterfly appearances in days for every kilometer increase in elevation (30 days for Rodopi and 16 days for Grammos). On the other hand, regressions for flight period duration in both regions had a negative slope (Grammos: −9.02, Rodopi: −10.06) and no significant differentiation emerged between mountains (LR test: 0.19, *p* = .67, *n* = 67), signifying a similar rate at which the duration of flight period changed with elevation. No significant differentiation in the rate of delay for butterfly assemblages across the three habitat types (Grammos LR test: 0.22, *p* = .91, *n* = 26; Rodopi LR test: 0.69, *p* = .74, *n* = 41) suggests that habitat type has little or no impact.

### 3.3 Phenological patterns at species level

We analyzed elevational patterns for 26 species: 20 had a positive slope when testing the relationship between the mean date and elevation, and six had a negative slope (Table 1). Significant slopes were positive for 11 species indicating a delay in the flight date with increase in elevation, and negative for one species indicating an opposite trend (Table 1). Of the species showing significant delays, *Pontia edusa* had the biggest delay (53.28 days/km) and *Polyommatus icarus* the smallest delay (10.57 days/km). The opposite pattern was seen for *Plebejus idas* (−20.21 days/km). Relationships between the duration of the flight period and elevation showed 20 negative and six positive slopes, out of which four were significant with negative relationship (Table 1). *Erynnis tages* had the steepest (57.37 days/km) negative slope or decrease of its flight period with elevation and *Melanargia galathea* had the smallest decrease (10 days/km) (Table 1). We found no interspecific evidence of effects on the species' elevational delay of the number of sites where species occurred (*p* = .98, *n* = 26) or the width of the species' elevational range (*p* = .40, *n* = 26).

**Table 1:** Results of the linear regressions for the mean date (days since 1 January, 1 January = 1) against elevation for the 26 selected species^a^

Species	Int_md_	*SE*_md_	Slope_md_ days/km	*P_md_*	Int_d_	*SE*_d_	Slope^d^ days/km	*P_d_*	No. of sites	Min alt.	Max alt.	Range (max–min)
*Aporia crataegi_u_*	143.48	6.14	24.89	***	25.48	3.42	−4.66	ns	17	128	1,516	1,388
*Argynnis paphia_u_*	182.97	5.42	13.90	**	11.92	4.41	−0.10	ns	26	128	1,410	1,282
*Aricia agestis*	155.93	21.00	38.55	*	41.34	14.20	−13.94	ns	12	406	1,453	1,047
*Brenthis daphne_u_*	144.25	12.27	29.67	*	23.91	3.52	1.73	ns	8	128	1,205	1,077
*Brintesia circe_u_*	170.05	4.89	22.40	***	12.96	1.77	−1.49	ns	12	420	1,638	1,218
*Coenonympha arcania_u_*	165.29	14.80	12.60	ns	27.39	27.84	−9.61	ns	7	860	1,410	550
*Coenonympha pamphilus*	184.13	9.84	−5.05	ns	22.71	6.88	10.47	ns	32	128	1,532	1,404
*Erynnis tages*	151.99	13.95	−5.00	ns	102.31	3.89	−57.38	*	5	406	1,247	842
*Iphiclides podalirius*	161.85	18.14	11.38	ns	22.33	11.03	5.06	ns	20	128	1,516	1,388
*Lasiommata megera*	185.33	9.90	−7.84	ns	36.55	9.14	−0.07	ns	8	128	1,746	1,618
*Leptidea duponcheli*	210.50	29.64	−4.55	ns	18.34	24.63	1.21	ns	8	252	1,745	1,493
*Leptidea sinapis*	174.48	13.18	20.80	ns	44.40	14.09	−18.18	ns	18	128	1,341	1,213
*Lycaena phlaeas*	159.47	12.93	27.59	*	42.16	13.38	−6.98	ns	17	128	1,458	1,330
*Lysandra bellargus*	172.67	48.23	44.51	ns	43.85	1.71	−23.33	**	6	128	1,188	1,060
*Maniola jurtina_u_*	154.38	5.76	37.72	***	22.64	3.39	−5.69	ns	20	128	1,532	1,404
*Melanargia galathea_u_*	167.25	2.91	23.62	***	24.16	4.17	−10.23	*	19	422	1,532	1,110
*Melitaea didyma*	160.88	10.28	13.78	ns	28.17	6.08	−8.13	ns	12	128	1,247	1,119
*Parnassius mnemosyne_u_*	121.09	13.88	29.75	ns	26.89	11.33	−5.07	ns	4	1,516	1,912	396
*Pieris mannii*	191.18	18.80	16.72	ns	28.90	3.21	−15.42	*	6	433	1,341	908
*Pieris rapae*	194.76	10.64	14.87	ns	23.35	8.49	−6.10	ns	17	128	1,458	1,330
*Plebejus argus*	191.68	−7.08	8.78	ns	16.88	7.10	5.83	ns	19	128	1,516	1,388
*Plebejus idas*	210.76	8.22	−20.21	*	13.85	6.76	10.81	ns	7	128	1,035	907
*Polyommatus icarus*	180.00	5.62	10.57	*	26.79	4.94	−9.53	ns	42	128	1,746	1,618
*Pontia edusa*	146.16	8.77	53.28	**	22.96	8.45	−6.10	ns	8	420	1,341	921
*Pyronia tithonus_u_*	221.25	41.27	−8.06	ns	37.07	3.28	−27.04	ns	4	546	1,035	489
*Thymelicus sylvestris_u_*	163.50	0.42	17.23	**	18.53	1.31	−8.37	ns	4	128	1,410	1,282

Note

Int: intercept, *SE*: standard error, subscript *md* corresponds to mean date and *d* to the duration of the flight period. Univoltine species are indicated by the subscript letter *u*, while the rest have more than one generation. Significance codes: 0 “***” 0.001 “**” 0.01 “*” 0.05 “ns” nonsignificant.

The species are in alphabetical order. The number of sites occupied, the minimum and maximum elevations (m), and the elevational range for each species are included too.

a Selected species are species recorded in more than three sites with at least two records per site and species overwintering as egg, pupae, or larvae, excluding thus early spring flyers and species overwintering as adults for which phenology may not be recorded comprehensively.

### 3.4 Temperature lapse rate

Considering both mountains and years, we found a significant decline of temperature with elevation of 3°C for every kilometer (*R* ^2^ = .42, *p* < .001, *n* = 67; mean temperature during sampling events = 24.8–3.2 × elevation). For Rodopi, mean temperature decreased by 3°C per kilometer in 2012 (*R* ^2^ = .34, *p* < .001, *n* = 41; mean temperature = 24.1–2.7 × elevation), and for Grammos, temperature decreased by 5°C per km in 2013 (*R* ^2^ = .63, *p* < .001, *n* = 26; mean temperature = 26.8–4.58 × elevation).

## 4 DISCUSSION

### 4.1 Date of appearance

In the two Mediterranean mountains studied, flight dates occurred later for butterfly communities at higher elevations, in agreement with the few previous studies of Mediterranean mountain butterfly communities (de Arce Crespo & Gutiérrez, 2011; Gutiérrez & Menéndez, 1998; Illán et al., 2012). The flight dates of individual species also generally occurred later at higher elevations (see also Forister & Shapiro, 2003). On the basis of a temperature lapse rate of approximately 3°C per every kilometer in elevation increase, our findings suggest that a 1°C decrease in mean seasonal temperature could be associated with a 4.66‐day phenological delay at the community level and an 7.71‐day (average) phenological delay at the species level. A similar trend of a 3.7‐day phenological delay for the entire butterfly community has been reported from Spain (de Arce Crespo & Gutiérrez, 2011; Illán et al., 2012).

The majority of univoltine species (70%) delayed the day of appearance with increasing elevation, whereas only 31.25% of the multivoltine species showed both delay and advance (Table 1). Overall, shifts in phenology for less flexible species, as the ones with a single annual reproductive cycle, are less pronounced than showed here (Macgregor et al., 2019). It is not, however, the first time where univoltine butterflies seem to be more prone to develop adaptations against the dry and hot summer of Mediterranean region (Garcia‐Barros, 1988). A potential butterfly strategy for increasing caterpillars' survival rate is to avoid the dry summer period and becoming more active on the cooler and wet months of early spring (Lopez‐Villalta, 2010).

On the other hand, previous work suggests that species with multiple generations may take advantage of warming conditions by increasing the number of generations (Altermatt, 2010) and thus showing thermal plasticity in life cycle regulation (Van Dyck et al., 2015). Greater synchrony in time of emergence across temperature gradients for multivoltine species has also been interpreted as a possible sign of adaptation to local climatic conditions (Roy et al., 2015). The altitudinal delays we observe are thus likely to be subject to plastic variation depending on annual climatic conditions. For example, Suggitt et al. (2012) found that butterflies occurring in both Britain and Catalonia can shift their use of different habitats or different local microclimates in response to year‐to‐year variation in climate. They concluded that species preferred the cooler conditions provided by closed habitats such as forests in hot years but were associated with warmer, more open habitats such as grasslands in cold years. Hence, although the altitudinal delay we observed for a species such as *Aporia crataegi* (24.89 days/km) was relatively close to the delay recorded in Spain with a similar approach (33 days/km, Illán et al., 2012), these rates are unlikely to represent fixed attributes of the species.

Because of caveats imposed by the space‐for‐time method such as the incapacity of tracking species responses in the long term or for detecting the effect of extreme weather conditions in successive summer periods, it is safer to follow the general trend implied by the slope and to interpret the observed patterns in the light of traits that make species susceptible to climate change. For example, *P. idas* (−20.21) was recorded earlier at higher elevation and cooler conditions. A possible explanation could be earlier availability of food resources at higher elevations: Similarly to butterflies, plants also have shortened their life cycles and advance their flowering and seed production as the climate has warmed (Steltzer & Post, 2009). Alternatively, negative species patterns might be regulated by an evolutionary adaptability to warmer climate, through an increased voltinism, despite the cooler local conditions at high elevation. An earlier appearance and prolonged flight period within areas above the timberline has also been reported for an alpine butterfly species (*Erebia epiphron*) in Czech Republic over the last decades (Konvička et al., 2016).

### 4.2 Duration of the flight period

Almost a 3‐day decline in the duration of the flight period for the community and an 8.86‐day average decline considering responses by individual species over elevation are in agreement with previous findings in the Mediterranean area (de Arce Crespo & Gutiérrez, 2011; Illán et al., 2012). We argue, however, that the lack of significant individual responses for most of the species tested is not simply due to a lack of statistical power, given that interspecific variability in elevational delays did not appear to be associated with sample size or elevational range.

Difference in species' diet spectrum might drive phenological changes at the level of individual species. We noticed that out of the four species that shifted their flight periods along elevation, three were woody feeders and only one was herbaceous plant feeder. According to Altermatt (2010), species feeding on herbaceous plants have smaller shifts in flight periods than the woody feeders because the second group has a narrow window to match the phenology to the flushing leaves. While herbs can produce leaves throughout the growing season, woody plants usually flush their leaves simultaneously (Feeny, 1976), and only for a short time of the year can accommodate the needs of herbivores for fresh and palatable resources. For the four species that exhibited a negative pattern in their duration of the flight period, three were multivoltine species and one was a univoltine species. In this context, the more pronounced phenological patterns for multivoltine life cycles are indeed a function of the species' voltinism.

### 4.3 Phenological patterns between mountains

The most striking feature is the inconsistency between elevational delays of butterfly assemblages between the two mountains with respect to temperature lapse rate. In particular, Rodopi showed a delay of 30 days for every kilometer increase in elevation compared to only 16 days in Grammos. However, the temperature lapse rate for Grammos in 2013 was −4.58°C/km, almost double that recorded in Rodopi in 2012 (−2.7°C/km). Indeed, Rodopi is located at higher latitude but in close vicinity to the sea, creating thus a mixture of Mediterranean and continental climate and a shallower temperature lapse rate, as opposed to Grammos which is located at the northwest edge of Pindos mountain range, where a mountainous continental climate prevails (Korakis, 2002; Xirouchakis, 2005).

A tempting explanation would be that the most abundant species that emerge later in the season drive the observed patterns, influencing altitudinal delays disproportionally (de Arce Crespo & Gutiérrez, 2011). However, this explanation is not valid in our case. The three most abundant species in Rodopi, *P. icarus* (407), *Coenonympha pamphilus* (351), and *C. crocea* (303) counting for 21% of the total records were present at both sites of low and high elevation (127–1,745 m; 127–1,458 m and 127–1,745 m, respectively) and had more than one broods covering the whole sampling period (from May to August), suggesting no such effect.

We argue that the steeper temperature lapse rate (5°C) along the elevation in Grammos may have driven species to better synchronize their activity resulting in a smaller delay overall. Empirical evidence suggests that populations from more variable environments have higher levels of plasticity which could preadapt them to extremes (Chevin & Hoffmann, 2017). When such extremes are lacking, it is logical to assume that species responses are not masked by phenotypic plasticity and therefore are steeper and more pronounced. Another explanation could be that species in Rodopi are closer to their upper thermal limits, and it is unlikely to evolve physiological tolerances to increased temperature (Araújo Miguel et al., 2013; Mills Simon et al., 2017). As a result, their performances are steeper and declines more pronounced compared to Grammos. Similarly, another study confirmed that for species adapted to high mean temperatures, it is more likely to experience detrimental phenological shifts to warmer climate (Scranton & Amarasekare, 2017). Further research to test the consistency of the patterns on each mountain and the establishment of permanent meteorological stations within each region might help to inform our findings.

### 4.4 Predictions and conclusions

On the basis of the different climatic scenarios proposed for the Eastern Mediterranean and Middle East, the mean temperature rise will be about 1–3°C in the near future (2010–2039), 3–5°C by mid‐century (2040–2069), and 3.5–7°C by the end of the century (2070–2099) (Lelieveld et al., 2013). Under the first scenario, based on the space‐for‐time substitution butterfly phenology would advance by 4.66–14 days in terms of the community and by 7.71–23.11 days for individual species; under the second scenario, the advances would be 14–23.33 days and 23.11–38.53 days, respectively, and under the third scenario, 16.33–32.66 days and 26.97–53.94 days. However, given the complexity and dynamism of the natural system, such radical changes of temperature are likely also to change many other contributing factors too, such as weather conditions at different times of year, as well as ecological community structure, where we are likely to see warm‐adapted species expanding at the expense of cold‐adapted ones (Zografou et al., 2014). A further aspect of system complexity is the ongoing forest encroachment that tends to counteract climate change, benefiting woodland species at the expense of others (Slancarova et al., 2016).

While it is difficult to foresee how organisms are going to cope under the ongoing changes in climate, it is possible that advanced emergences could threaten serious trophic disruption between interacting groups. Our findings both confirm an earlier and prolonged activity at lower elevations overall. At the same time, confound expectations for ectotherms such as signs of earlier appearance in high elevations for multivoltine organisms and more pronounced shifts in flight periods for the woody feeders challenge the idea that these species assemblages have special thermal traits that confer adaptive advantage under new conditions.

## CONFLICT OF INTEREST

None declared.

## AUTHOR CONTRIBUTIONS

VK, JMH, RJW, AG, and KZ conceived and designed the experiments. KZ performed the experiments, and KZ and GCA analyzed the data. KZ wrote the first draft of the manuscript, and AG, RJW, JMH, VK, and GCA contributed to subsequent versions of the article and agreed on the final version to be published.

### Open Research Badge

This article has earned an Open Data Badge for making publicly available the digitally‐shareable data necessary to reproduce the reported results. The data is available at https://datadryad.org/stash/share/0wyAaQClpdviKiC3L8Ufg_U0pOcHfHVX8PwK9tUfKI0.

## Data Availability

Data connected to the manuscript with the title “Butterfly phenology in Mediterranean mountains using space‐for‐time substitution” have been deposited in a publicly accessible repository Dryad: https://datadryad.org/stash/share/0wyAaQClpdviKiC3L8Ufg_U0pOcHfHVX8PwK9tUfKI0.
