# Supplementary material for: Butterfly phenology in Mediterranean mountains using space‐for‐time substitution
Source: Ecol Evol. 2020 Jan 2;10(2):928–39. doi: 10.1002/ece3.5951 (PMC6988524; doi:10.1002/ece3.5951)
Supplement: Supplementary file 1 [file ECE3-10-928-s001.docx]

**Supporting Information**

Title: Butterfly phenology in Mediterranean mountains using space-for-time substitution

Konstantina Zografou^1, 2^, Andrea Grill^1^, Robert J. Wilson^3^, John M. Halley^2^, George C. Adamidis^1^, Vassiliki Kati^2^

^1^Institute for Ecology and Evolution, University of Bern, Baltzerstrasse 6, CH-3012, Bern, Switzerland

^2^ Department of Biological Applications and Technology, University of Ioannina, 45110, Ioannina, Greece

^3^ Museo Nacional de Ciencias Naturales (MNCN-CSIC), Madrid E28006, Spain

Corresponding author: Konstantina Zografou

E-mail: [konstantina.zografou@iee.unibe,ch](mailto:konstantina.zografou@iee.unibe,ch)

**
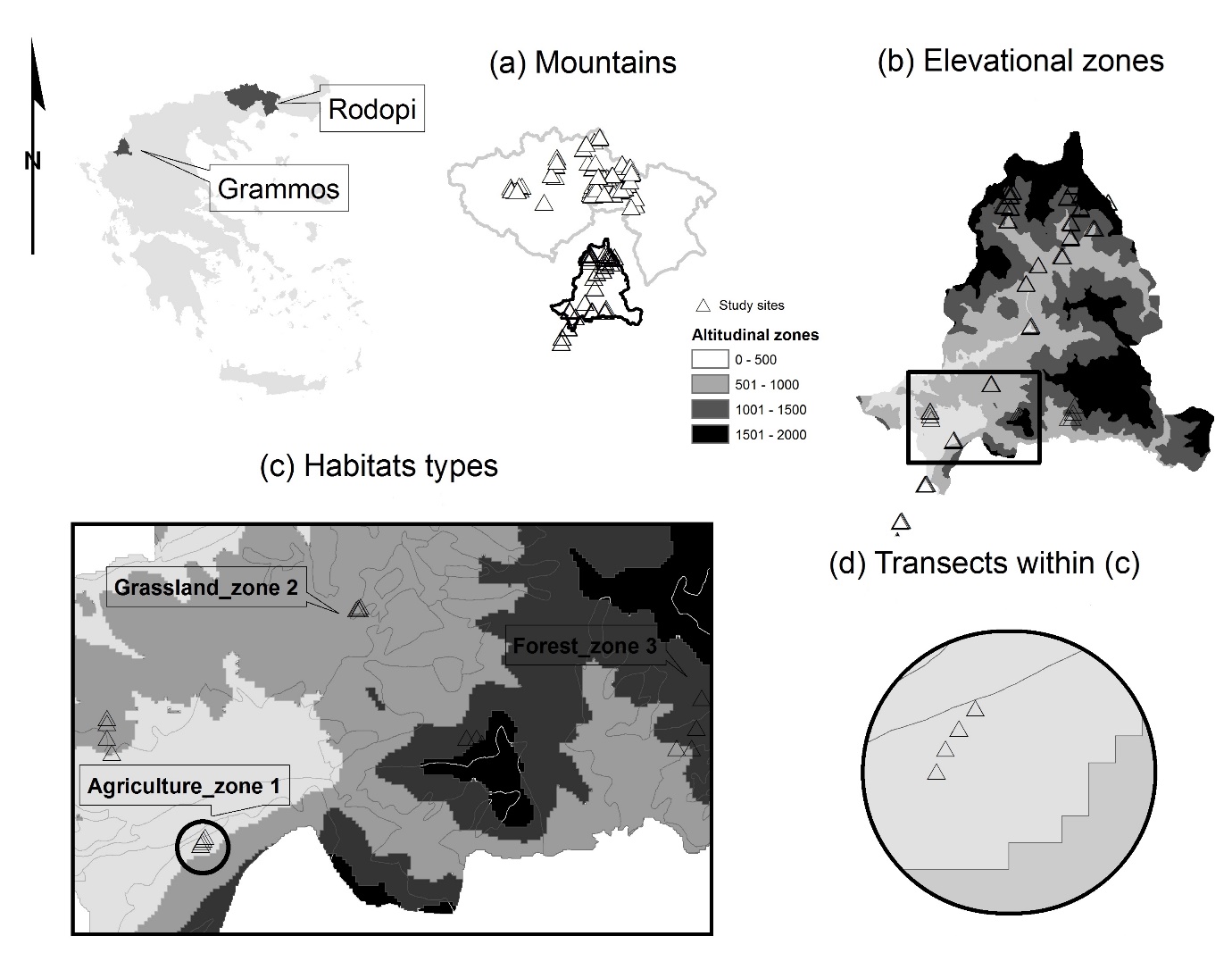
**

**Figure S1.** Study area and nesting sampling design across four spatial scales (a-d). Scales are: (a) two mountains, (b) four elevational zones, (c) three habitat types, (d) 67 transects (or sites). The grey gradient in (c) corresponds to the four elevation zones and each polygon is assigned to one of the three habitats studied (agriculture areas, forests, grasslands).

**Table S1**. Sampling design: proportions (%) of Corine cover and transects per elevational zone, habitat type and mountains; where Zone 1= 0-500m, Zone 2= 501-1500m, Zone 3 =1001-1501m, Zone 4= 1501-2000m. Inside the parenthesis is the actual number of sites (replicates).

|  |  | Grammos | |  | Rodopi | |
| --- | --- | --- | --- | --- | --- | --- |
|  |  | Corine cover | Sites |  | Corine cover | Sites |
| Zone 1 | Agriculture | 40.24 | 33.33 (2) |  | 51.42 | 38.46 (5) |
|  | Forests | 37.8 | 33.33 (2) |  | 24 | 46.15 (6) |
|  | Grasslands | 20.73 | 33.33 (2) |  | 17.37 | 15.38 (2) |
| Zone 2 | Agriculture | 4.8 | 33.33 (2) |  | 8.89 | 20 (2) |
|  | Forests | 62.66 | 33.33 (2) |  | 66.06 | 50 (5) |
|  | Grasslands | 31.81 | 33.33 (2) |  | 24.74 | 30 (3) |
| Zone 3 | Agriculture | 1.19 | 25 (2) |  | 0.38 | 18.18 (2) |
|  | Forests | 68.95 | 25 (2) |  | 79.84 | 27.27 (3) |
|  | Grasslands | 29.85 | 50 (4) |  | 19.64 | 54.54 (6) |
| Zone 4 | Agriculture | 0 | 0 (0) |  | 0 | 0 (0) |
|  | Forests | 43.75 | 28.57 (2) |  | 57.39 | 28.57 (2) |
|  | Grasslands | 56.25 | 71.42 (5) |  | 42.6 | 71.42 (5) |

**
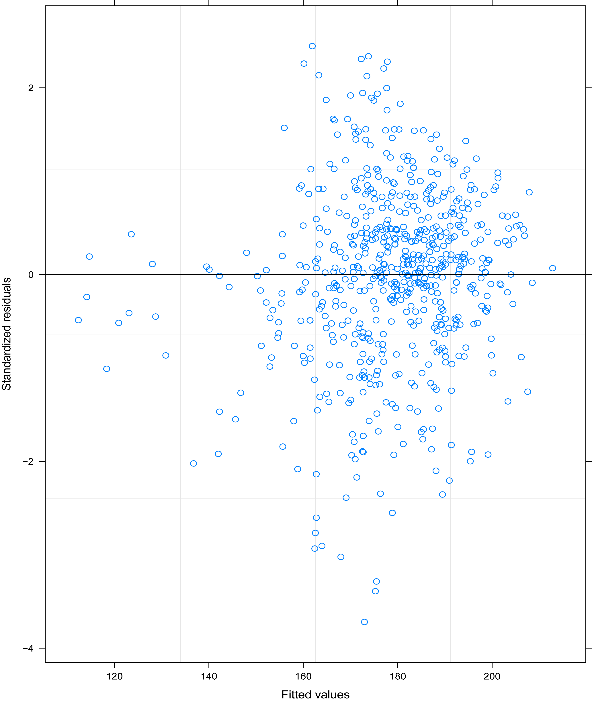
**

**
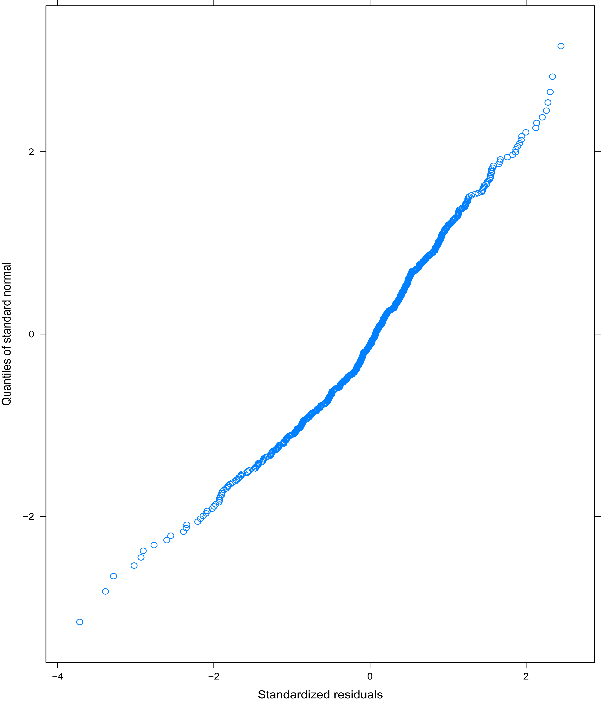
**

**
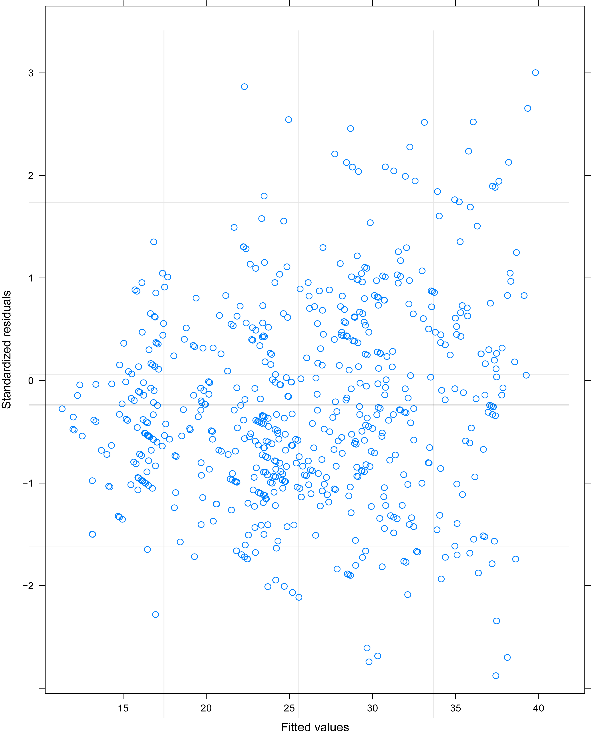
**

**
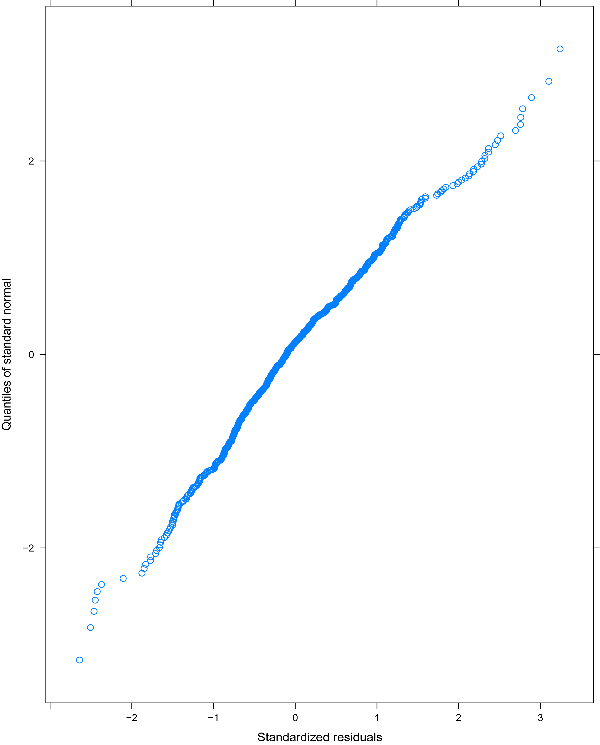
**

**Figure S2.** Diagnostic plots for the linear mixed models where the mean date and standard deviation about the mean date were modelled as a function of altitude, mountain and habitat. In addition, species were included as a random effect; homoscedasticity of the residuals for the mean date of appearance (upper left panel) and duration of the flight period (lower left panel) and normality of the residuals of the mean date of appearance (upper right panel) and duration of the flight period (lower right panel).
